# Women and entrepreneurship for economic growth in Indonesia

**DOI:** 10.3389/fpsyg.2022.975709

**Published:** 2023-01-09

**Authors:** Retno Purwani Setyaningrum, Nor Norisanti, Mochammad Fahlevi, Mohammed Aljuaid, Sandra Grabowska

**Affiliations:** ^1^Department of Management, Universitas Pelita Bangsa, Bekasi, Indonesia; ^2^Department of Management, Universitas Muhammadiyah Sukabumi, Jawa Barat, Indonesia; ^3^Department of Management, BINUS Online Learning, Bina Nusantara University, West Jakarta, Indonesia; ^4^Department of Health Administration, College of Business Administration, King Saud University, Riyadh, Saudi Arabia; ^5^Department of Production Engineering, Silesian University of Technology, Gliwice, Poland

**Keywords:** servant leadership, E-commerce digitalization adoption, self-efficacy, women, entrepreneurship

## Abstract

Business and entrepreneurship are certainly not a monopoly on men. As inhabitants of half the world, women also have the right to engage in the business world. In line with the ideals of emancipation, many women currently have the opportunity to become business partners of men. This research aims to clarify the role of absorptive capacity and entrepreneurial competencies in strengthening women’s leadership toward success. This research was conducted in 3 provinces in Indonesia involving 114 women entrepreneurs in the MSME (micro, small, and medium enterprises) category. This research used a structural equation model with the SmartPLS software tool. The results of this research showed that absorptive capacity and entrepreneurial competencies were able to increase women’s leadership toward successful entrepreneurship. City and age moderation failed to moderate the effect of women’s leadership on successful entrepreneurship. The government on a macro scale needs to pay attention to providing understanding or special training for women MSME business actors who are trying to build their business from scratch. Business challenges for women entrepreneurs are heavier than for men because there are several social, value, and cultural barriers that require special treatment and strategies in developing the potential of women entrepreneurs in Indonesia.

## Introduction

Economic growth in Indonesia is relatively slow. It can be seen from unemployment as a major problem in Indonesia (Desky et al., 2020). The number of the labor force is inversely proportional to the number of jobs available. The formal sector is still the target of the community in choosing a job. Thus, people do not try to create their own jobs in the non-formal sector or the private sector when the formal sector is sluggish. It makes the unemployment rate is quite high in Indonesia (Fahlevi et al., 2020; Pritadrajati et al., 2021). According to Ataei et al. (2020) based on McClelland’s theory, a country is considered to be prosperous if at least 2% of its population are entrepreneurs. The solution is to increase the interest in entrepreneurship of the younger generation (Leong et al., 2022). Therefore, it can overcome unemployment and improve the Indonesian economy.

Poor people have caused various excesses in people’s lives, such as job opportunities (Prasetyo et al., 2022). The number of jobs added is not accompanied by a high population. People have difficulty finding jobs. The various fields of work available are not able to accommodate a large number of workers. The number of job seekers is increasing day by day. It is not matched by the availability of job opportunities. Limited employment opportunities lead to intense competition in job selection. A small part of the workforce with qualified skills is accepted, while other members of the community do not find work and even become unemployed. If people get a job, it is not feasible or does not follow their abilities (Adnani et al., 2022).

The world of business and entrepreneurship is certainly not a monopoly of men (Halilem et al., 2022). As inhabitants of half the earth’s surface, women have the right to also engage in the business world (Chatterjee et al., 2022). In line with the ideals of emancipation, many women have the opportunity to become business partners of men. Due to the development of an increasingly globalized era, women are also affected by globalization which in turn makes them more equal and increasingly shows the same abilities as men, or even exceeds them. This welfare also dispels the myth that has been aimed at women so far (Discua Cruz et al., 2022). It is enough for them to stay at home to do their routine tasks as wives and housewives, such as cooking, decorating themselves and giving birth, and taking care of children. The mythical role and nothing more than prejudice are clearly not communicative in everyday life, because women are human resources that are no less important in completing various aspects of life. It should be recognized that the status of women in several foreign countries is categorized as second-class citizens who do not deserve the same rights as men. Women are also always sidelined in various matters, such as in politics, work, and business. The existence of women is a perfect complement to God’s creation, even though they are companions and partners of men in all things (Handaragama and Kusakabe, 2021).

In line with the issue of entrepreneurship, although there is no detailed data regarding the number of female entrepreneurs, the problems can be ascertained to be the same (Webster and Haandrikman, 2017). In this regard, the research on the success factors of women entrepreneurs becomes interesting. The underlying argument is that the number of women tends to be more than men. Demographically, the role of women entrepreneurs can support improving family welfare besides their important role in labor absorption and also reducing poverty in the region (Varela-Candamio et al., 2018). Another interesting factor related to the research of women’s entrepreneurship is the identification of the driving factors, it is the push and pull factors. Therefore, gender identification of entrepreneurial success factors provides an opportunity for comparative studies (Ramadani et al., 2022). In addition, findings about the success factors of women entrepreneurs in the majority of developing countries tend to be influenced by the “push” factor compared to the “pull” factor (Dheer et al., 2019; Guzman and Kacperczyk, 2019; Figueroa-Domecq et al., 2020). Human resources, especially women, have many limitations. They are related to opportunities and customary norms. There is a group of women who do not obtain better opportunities than other human resources because they are not bound by some of the above limitations. Therefore, it is very important to pay attention to women’s human resources. Hence, in the future, the next generation of Indonesian youth will be better. Encouragement, encouragement of knowledge and skills as well as other incentives that can lead to women’s empowerment is very much needed.

The support of various studies that shows women’s entrepreneurial abilities are superior to men as shown in the findings of research by Hendratmi et al. (2022) that women are superior in multitasking (doing several jobs at once). Hence, women can do several jobs at the same time. As nature as a woman and a mother, women are used to doing several jobs at almost the same time. Thus, there is no reason for women to not be able to do anything. All it takes is determination and the will to give it a try. The purpose of doing business is for what and who. When they have found the reasons and goals for doing business, the determination to achieve them is even greater. The multitasking ability of women is the main capital in running the business (Eger et al., 2022). This means that women have a great opportunity to succeed in business. Maity and Barlaskar (2022) explained that women have leadership abilities in several aspects that are better than men, although this is still a heated debate.

There are many abilities of women related to business that can be developed. When running a business, marketing is certainly needed. Generally, women are more flexible in communicating and introducing something new to others (Leung et al., 2022). Women are better able to adjust the conversation quickly. This eases a woman to do business and introduce her products to potential buyers. With these abilities, especially in their communication, the items offered are easier to promote and sell. In addition, women are also decision-makers. They are sometimes the decision makers in their household from small things to big things. It can be considered that women are always involved in making decisions in the household (Kochar et al., 2022). Women take a very important role in the process alongside their husbands. That means, if women become business people, women can also be successful because they are used to being decision makers (Saleemi and Kofol, 2022). Women are generally more sensitive to the needs of their families today. They can represent themselves for every product that will be sold or service that will be offered later in demand by many people or not. Women better understand consumer desires because they generally apply to themselves if they are consumers when they want to buy the products or services offered. In business, making decisions quickly is very important. Women are also used to this kind of thing. So, there is no reason that women do not fit for business (Liu et al., 2021).

The increasing participation of women in the business sector is a worldwide phenomenon (Liu et al., 2021). In Asia, 35% of small and medium enterprises (SMEs) are led by women. 25% of new businesses in China are run by women. In Japan, four out of five SMEs are owned by women. This phenomenon also occurs in Indonesia. Although there is no definite data that can be obtained, the number of women entrepreneurs in Indonesia is quite large. As an illustration, since its establishment on February 10, 1975, the Indonesian Women Entrepreneurs Association (IWAPI) has had 15,000 members spread across all provinces in Indonesia. Therefore, most are small and medium entrepreneurs (97%) and only 3% are large entrepreneurs (IWAPI, 2022).

The world of business and entrepreneurship is certainly not a monopoly of men. As inhabitants of a half-world, women have the right to also engage in the business world (Liu et al., 2021). In line with the ideals of emancipation, many women have the opportunity to become business partners of men. Due to the development of an increasingly globalized era, women are also affected by globalization which in turn makes women more equal and increasingly shows the same abilities as men (Ladge et al., 2019). Nowadays, there are many successful businesswomen both in their businesses and in their families. There is nothing wrong with adding or helping the family income. This has been conducted by many women today (Dheer et al., 2019; Eger et al., 2022; Hendratmi et al., 2022). If two people have income in the family it will be better. Indeed, many women experience a dilemma in running their careers and their families at the same time. Many think that women have a career so the family will be neglected. In fact, this is not the case because many facts show that women are able to succeed in both. The advantage of running a business at home, women can still take care of the children and their working hours are more flexible than office work. Thus, women will add value to the family (Fine and Sojo, 2019).

Statistics have shown positive developments for women regarding life expectancy, gender empowerment index, and even retail investment figures. Furthermore, the survey proves that the proportion of women in strategic positions in companies continues to grow (Bruce et al., 2022). There is a projected additional world GDP of 28$ trillion if there is gender equality. This positive thing further defines the role of women as naturally born leaders who hold the balance in the professional world to the household (Sheerin and Garavan, 2022). This phenomenon raises significant questions and research gaps about whether women can properly sustain the businesses they lead. Women are invited to redefine their leadership and determine the definitive direction they want for themselves without being affected by societal stigma or family demands that hold conservative views because anyone should be able to become a leader, both themselves and family leaders, as long as they can carry out with real steps (Franczak and Margolis, 2022).

The success of women entrepreneurs is inseparable from the encouragement of economic and family needs as well as opportunities such as self-confidence and strong affection. The indicators in women leadership can have an impact on becoming successful women entrepreneurs (Ladge et al., 2019; Discua Cruz et al., 2022; Hendratmi et al., 2022). Good leadership behavior and moral leadership are significantly related to employee creativity which indirectly affects the success of an entrepreneur (Bruce et al., 2022; Franczak and Margolis, 2022; Sheerin and Garavan, 2022). The existence of research on women’s leadership and the success of women entrepreneurs still has significant and insignificant results (Fine and Sojo, 2019; Bruce et al., 2022; Maity and Barlaskar, 2022; Sheerin and Garavan, 2022). thus, it requires another mediating role that can be considered in the research model.

A key encouragement for entrepreneurship research is its relationship to business success and its contribution to economic development. A question that remains in the literature is what distinguishes the women entrepreneurs who eventually develop their companies (Wilson and Newstead, 2022). In this research, women entrepreneurs are defined as business leaders who are owned and managed by women wholly or in the majority (Liu et al., 2021; Discua Cruz et al., 2022). Existing literature suggests that women entrepreneurs tend not to focus on early and rapid growth (Ladge et al., 2019), but prefer in some cases to remain small (Hendratmi et al., 2022). However, the notion of failure of success among women entrepreneurs has been challenged (Fine and Sojo, 2019). In this regard, some authors argued that in pursuing success, women entrepreneurs place less emphasis on measurable criteria. A theoretical model is built and tested to include different aspects of the three broad antecedents of corporate growth such as capabilities, needs, and opportunities. This research focuses on entrepreneurial competence and absorptive capacity, both of which are related to ability (Dawa et al., 2020).

The capabilities needed to grow a company can be conceptualized in several methods (Bibu et al., 2016). We adopted the concept of entrepreneurial competence as a way to capture the knowledge, skills, and abilities required for business success (Mitchelmore and Rowley, 2013). Kamuri (2021) defined entrepreneurial competence as the entrepreneur’s total ability to perform job roles successfully. Competence shapes the aspirations and choices of entrepreneurs about the direction of the company. They are also useful in mobilizing and converting other types of resources for company expansion (Namatovu and Dawa, 2017). They are critical to securing scarce resources for women entrepreneurs in Indonesia, who face relatively higher institutional challenges in this sociocultural context.

Over the last few decades, there has been a significant increase in the number of women entrepreneurs worldwide (Liu et al., 2021). Malaysia is a neighboring country that has the same social and demographic values of society as Indonesia. According to Mamun et al. (2017) in Malaysia, women’s participation in economic activities began in 1970, wherein in Peninsular Malaysia, 18 percent of the female workforce were classified as “self-employed,” while 2.3 percent were classified as employers and 13 percent were workers labeled as “wholesale and retail trade employer.” This clearly shows that many women are involved and show great interest in entrepreneurship. As noted in a recent survey by the Department of Statistics, Malaysia, most women are actively involved in entrepreneurial activities and own 19.7 percent of Malaysia’s total SMEs. 91.7 percent of them are engaged in the service sector and a small portion of 6.9 percent are engaged in manufacturing, and the remaining 1.4 percent are active in the mining, agriculture, and construction sectors. This shows the active participation of women in entrepreneurship considering that 99 percent of companies in Malaysia are SMEs and they contribute 31 percent of the national GDP due to the contribution of women micro-entrepreneurs, and the lack of studies among female micro-entrepreneurs (Mamun et al., 2017).

Entrepreneurial competence is acquired through previous work experience, formal studies, and prior business ownership (Khalid and Bhatti, 2015). Women face many gender-based barriers in these activities, including discrimination in the workplace, exclusion from pursuing certain study programs due to cultural norms, and lack of capital to start a business (Webster and Haandrikman, 2017). While research has assessed that lack of competence is a critical barrier to success among women entrepreneurs (Dawa et al., 2020), studies explaining this relationship are few with calls for further research to improve our understanding of this relationship. In fact, mixed results have been noted in assessing the relationship between competence and success, suggesting the possibility of a more explanatory variable. Therefore, this research seeks to explain what we propose as a distal relationship between leadership and the success of women-owned firms by explaining the mediating role of absorptive capacity and entrepreneurial competence.

The resource-based view explains the inimitable role of resources in firm performance. Previous research had identified various types of resources that are important for the realization of the growth of women-owned enterprises (Maity and Barlaskar, 2022; Sheerin and Garavan, 2022). We seek to add absorptive capacity to this rich line of inquiry by bridging the gap between women’s leadership and performance outcomes. This research showed that while competence was an important selection criterion, absorbency provides more clarity in explaining business success. Based on this background, this research aims to explain the key factors of the success of women entrepreneurs in Indonesia.

## Methodology

This research was conducted using a quantitative approach to determine the causal relationship between variables (Lind et al., 2018). The population in this research was women entrepreneurs of MSME in three provinces in Indonesia, namely West Java, Yogyakarta, and Jakarta. These three provinces were chosen because the economic growth in these three provinces was the highest compared to other provinces in Indonesia (Badan Pusat Statistik, 2019).

West Java is one of the engines in developing its creative economy at the national level. The creative economy plays a major role in the economy of West Java and even nationally because it is supported by a large population and supporting facilities. Business products MSME such as culinary to fashion from West Java are also widely recognized by the global market. The rapid growth of the creative economy has made West Java Province the largest contribution to national exports in January–June 2021 which reached US $16.08 billion or 15.63 percent of total national exports. Yogyakarta has many culinary, craft, and fashion businesses which are the three mainstay MSME products that the Yogyakarta local government has begun to reorganize to attract tourists again. The business sector is still the main support for tourism activities in Yogyakarta. The Yogyakarta government records that there are 63.9 million micro-enterprises that already have business legality in the form of a business registration number. Jakarta has the privilege of being the nation’s capital and center of government, as well as the center of economy, business, and services, including the center of investment. As the center of the business and tourism economy, where millions of people try their luck in search of a decent living, Indonesia’s current economic turnover is more than 50 percent in Jakarta (Badan Pusat Statistik, 2019).

The sampling technique in this research was proportional area sampling by involving respondents in each province and using the purposive sampling technique. The criteria used were (1) MSME with a minimum sales turnover of 5 million per month, (2) women entrepreneurs with a minimum of high school education, (3) MSME that have been operating for at least 2 years, and (4) MSME have been officially registered by the Ministry of Cooperatives and MSME. This research collected data with online and offline questionnaires within 3 months of data collection. The results of collecting data from respondents in this research amounted to 127 respondents with the following details: (1) West Java with 37, (2) Yogyakarta with 38, and (3) Jakarta with 52. We conducted an online and offline questionnaire survey from April 2022 to June 2022 for women entrepreneurs belonging to MSMEs, the Snowballing sampling method was also adopted in the offline channel. A total of 127 participants filled out the questionnaire. Three types of responses were screened: First, those who were not leaders or business owners (121); Second, those who filled out the questionnaire took less than 2 min (118); Third, the results of filling out all items have almost the same value (114). After filtering poor data, there were 114 valid questionnaires.

The measurement of construct of women’s leadership is measured by the Likert scale adopted from the research conducted by Pawirosumarto et al. (2017) consisting of leader-member orientation, task structure, and position power. The construct of absorptive capacity was measured by a Likert scale adopted from Dawa et al. (2021). It consists of acquisition, assimilation, transformation, and exploitation. The construct of entrepreneurial competencies was measured by a Likert scale adapted from the research of Bamiatzi et al. (2015) on entrepreneurial, managerial, personal, and human relationships. The successful entrepreneur construct is measured by the Likert scale adapted from the research of Asandimitra and Kautsar (2017) consisting of serving people, being creative, competitive, and developing and implementing new ideas.

SmartPLS was adopted to analyze measurements and structural models with partial least square structural equation modeling (PLS-SEM), which has a better predictive ability with relatively small sample sizes. To test the structural model, 5,000 subsamples, bias correction, and acceleration were prepared in the PLS bootstrap algorithm (Hair et al., 2017). The two-tailed hypotheses testing was performed.

## Results and discussion

### Measurement of model assessment

**FIGURE 1:**
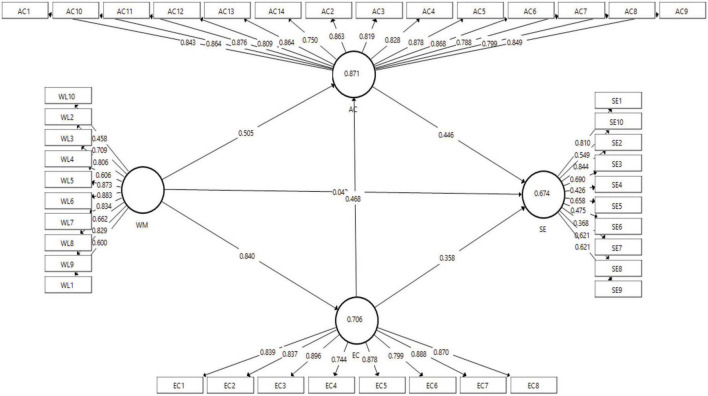
Structural model.

In the beginning, the outer loading measurement was conducted to obtain the right measurement for each construct in the model. This measurement was taken from data that had been collected through a questionnaire with a Likert scale of 1–5. The measurement of the outer model was calculated on the PLS Algorithm to find out the amount of outer loading on each item with the construct. Thus, if there are items that are still below 0.5 then they would be deleted from the model. The AVE value had to meet the requirements of 0.5. Cronbach’s alpha and composite reliability were not allowed below 0.7 (Hair et al., 2017). The structural model can be seen in Figure 1.

Figure 1 shows that women’s leadership is measured by 10 items, absorptive capacity is measured by 14 items, entrepreneurial competencies are measured by 8 items, and successful entrepreneurship is measured by 10 items. In Table 1, the calculation results from the PLS Algorithm are explained in detail as follows:

**TABLE 1:** Convergent validity and reliability.

Variable and item	AVE	Outer loading	Cronbach’s alpha	Composite reliability
Women leadership	0.545		0.902	0.921
• WL1		0.600		
• WL2		0.709		
• WL3		0.806		
• WL4		0.606		
• WL5		0.873		
• WL6		0.883		
• WL7		0.834		
• WL8		0.662		
• WL9		0.829		
• WL10		0.458		
Absorptive capacity	0.699		0.967	0.970
• AC1		0.843		
• AC2		0.863		
• AC3		0.819		
• AC4		0.828		
• AC5		0.878		
• AC6		0.868		
• AC7		0.788		
• AC8		0.799		
• AC9		0.849		
• AC10		0.864		
• AC11		0.876		
• AC12		0.809		
• AC13		0.864		
• AC14		0.750		
Entrepreneurial competences	0.715		0.942	0.952
• EC1		0.839		
• EC2		0.837		
• EC3		0.896		
• EC4		0.744		
• EC5		0.878		
• EC6		0.799		
• EC7		0.888		
• EC8		0.870		
Successful entrepreneur	0.389		0.871	0.858
• SE1		0.810		
• SE2		0.844		
• SE3		0.690		
• SE4		0.426		
• SE5		0.658		
• SE6		0.475		
• SE7		0.368		
• SE8		0.621		
• SE9		0.621		
• SE10		0.810		

**FIGURE 2:**
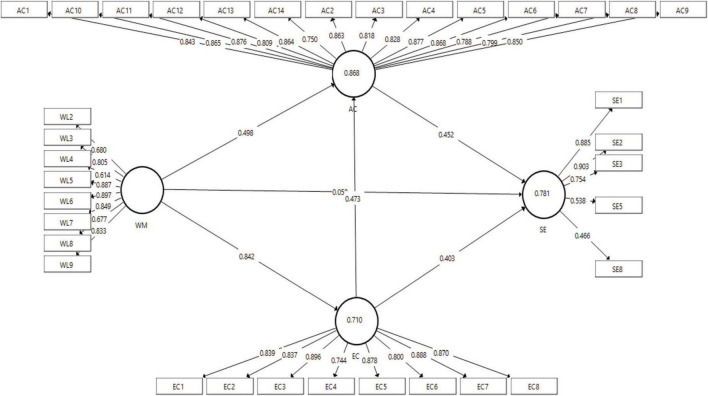
Structural model modification.

In Table 1, it is known that several indicators do not meet the standard values, such as the outer loading value which is still below 0.5 and some constructs have an AVE value below 0.5 so it is necessary to modify the model by removing several items that do not meet these requirements before path analysis is carried out the analysis. Figure 2 is the final result of the modification of the structural model:

In Figure 2, it is known that there are several deleted items because they did not meet the standard values, especially in the constructs of women’s leadership and successful entrepreneurship. Thus, after being deleted, it left several items that were valid and met the requirements, such as after modifying the model, the outer loading value and the AVE value increase in both constructs, the modification of this model will be able to support precise measurements during path analysis later. In Table 2, the results of changes in validity and reliability are summarized after modification of the model.

**TABLE 2:** Convergent validity and reliability (modification).

Variable and item	AVE	Outer loading	Cronbach’s alpha	Composite reliability
Women leadership	0.619		0.909	0.928
• WL2		0.680		
• WL3		0.805		
• WL4		0.614		
• WL5		0.887		
• WL6		0.897		
• WL7		0.849		
• WL8		0.677		
• WL9		0.833		
Absorptive capacity	0.699		0.967	0.970
• AC1		0.843		
• AC2		0.863		
• AC3		0.819		
• AC4		0.828		
• AC5		0.878		
• AC6		0.868		
• AC7		0.788		
• AC8		0.799		
• AC9		0.849		
• AC10		0.864		
• AC11		0.876		
• AC12		0.809		
• AC13		0.864		
• AC14		0.750		
Entrepreneurial competences	0.715		0.942	0.952
• EC1		0.839		
• EC2		0.837		
• EC3		0.896		
• EC4		0.744		
• EC5		0.878		
• EC6		0.799		
• EC7		0.888		
• EC8		0.870		
Successful entrepreneur	0.535		0.785	0.845
• SE1		0.883		
• SE2		0.902		
• SE3		0.752		
• SE5		0.544		
• SE8		0.572		

Discriminant validity was carried out to ensure that each concept of each construct in the model is different from other constructs. Discriminant validity testing was conducted to find out how precisely a measuring instrument performs its measurement function. In Table 3, discriminant validity was tested using the Fornell-Larcker Criterion.

**TABLE 3:** Discriminant validity (Fornell-Larcker Criterion).

Constructs	AC	EC	SE	WM
AC	0.836			
EC	0.781	0.845		
SE	0.732	0.821	0.872	
WM	0.789	0.802	0.801	0.820

The results in Table 3 show that there was no cross-loading symptom in each construct in the model because the loading on each construct has the largest value compared to measurements on different variables.

### Path analysis

**FIGURE 3:**
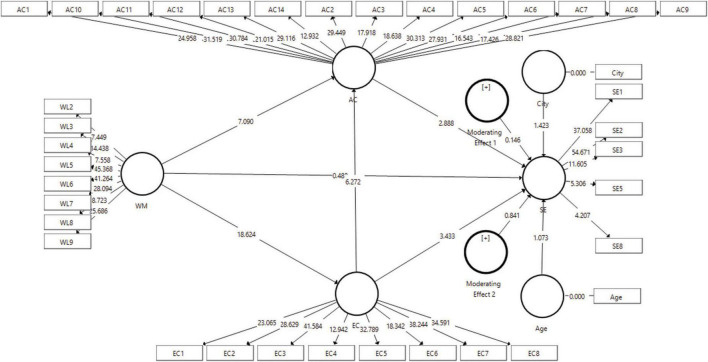
Bootstrapping.

At the end of the statistical calculation, bootstrapping calculations are conducted to determine the effect on each construct in the model according to the direction of the analysis path. In the final model, bootstrapping is carried out according to Figure 3 using 2 moderating variables, City and Age.

In Figure 3, it can be seen that bootstrapping was conducted on 4 latent constructs and 2 moderating constructs that function as moderators on the WM to SE path. In Table 4, it is known that the details of the bootstrapping results are as follows:

**TABLE 4:** Path coefficients estimation.

Hypothesis	Variables	*t*-statistics	*p*-values	Supported
H1	WM → SE	0.489	0.625	No
H2	WM → AC	7.046	0.000	Yes
H3	WM → EC	18.664	0.000	Yes
H4	AC → SE	2.878	0.004	Yes
H5	EC → AC	6.249	0.000	Yes
H6	EC → SE	3.429	0.001	Yes
H7	WM → AC SE	2.607	0.009	Yes
H8	WM → EC SE	3.443	0.001	Yes
H9	WM → SE → (AGE)	0.842	0.400	No
H10	WM → SE → (CITY)	0.146	0.884	No

**FIGURE 4:**
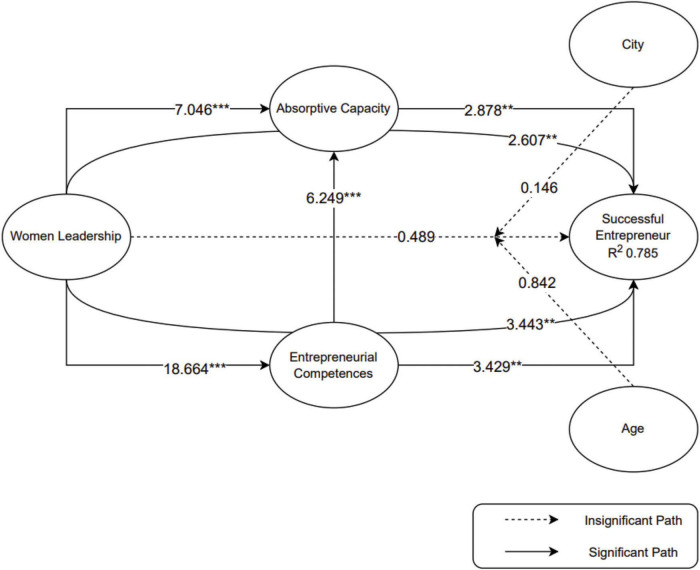
Path significance (***p < 0.001, **p < 0.01, *p < 0.05).

In Table 4, it is known that almost all path analyses have significant results so most of the hypotheses in this model are accepted, while only 3 hypotheses are rejected, namely H1, H9, and H10. Figure 4 will explain the significant and non-significant paths to ease in observing.

In Figure 4, the quality of the endogenous construction of the structural equation model could be measured by the value of R2 which had three thresholds of 0.75, 0.50, and 0.25, representing strong, medium, and weak predictive accuracy (Lind et al., 2018). In the SE endogenous variable, it was known that the R2 value was 0.785. thus, it was quite strong because it could explain 78.5% of endogenous constructs in this model.

## Discussion

This research found that there was a weak effect between women’s leadership on successful entrepreneurship. Thus, there were insignificant results. This result cannot be separated from Indonesian culture which views it as an inappropriate gender to lead. This stigma has been attached for a long time as part of society’s values which view that a woman should be a housewife, stay at home, and not as a breadwinner for the family. However, women’s leadership often shows different leadership and different traits in different environments, such as male-dominated environments where women are considered more assertive and autocratic (Sharif, 2019). How women leaders apply their leadership style also depends on the socio-cultural environment (Maity and Barlaskar, 2022). Current research also found that women try to achieve individual success in male-dominated environments by turning to a masculine approach and distancing themselves from other women (Dawa et al., 2020; Bruce et al., 2022; Franczak and Margolis, 2022; Wilson and Newstead, 2022).

Absorptive capacity is known to be able to mediate the effect of women’s leadership on successful entrepreneurship. Successful entrepreneurs can learn and link learning to their current knowledge base. The main premise is, that knowledge is cumulative, so the more an individual or organization knows, the easier it is to acquire new knowledge. Prior knowledge is considered essential for the assimilation of new knowledge, which means to say that individual and organizational learning capacities depend on pathways as outcomes of previous learning efforts and abilities (Dawa et al., 2020). Women leadership requires a dynamic and cumulative process, in the sense that knowledge gained in the present will be more efficiently accessed in the future (Kim and Park, 2018). Absorptive capacity provides a new perspective on women’s leadership in the businesses they run, especially in the context of MSMEs that are not structured like large companies (Webster and Haandrikman, 2017; Handaragama and Kusakabe, 2021). A woman’s leadership who has an openness and the ability to absorb external benefits in sustainability learning is proven to be able to strengthen her influence in leading and running a business toward success (Mitchelmore and Rowley, 2013).

Previous studies have found that absorption was positively related to innovation and business success (Mamun et al., 2017). A high level of absorption is able to produce better innovation performance (Namatovu and Dawa, 2017; Dawa et al., 2020). Constantly scanning the environment for external knowledge and leveraging enables companies to better cope with the dynamic economy and market environment and remain innovative by producing and providing products and services that meet the needs of emerging markets (Vo, 2016). Therefore, this research postulated that absorption capacity leads to improved performance and success of MSMEs, which ultimately leads to better economic growth in Indonesia (Fahlevi and Alharbi, 2021; Fahlevi et al., 2022; Hendratmi et al., 2022).

Entrepreneurial competencies are key factors for business success according to previous research (Mitchelmore and Rowley, 2013). Entrepreneurial competencies are very relevant to the successful implementation of entrepreneurship. An entrepreneur is considered to be competent when they have the ability to find and effectively use the necessary resources to achieve business goals (Ladge et al., 2019; Discua Cruz et al., 2022). In the context of Indonesia, MSMEs led by women tend to be poorly structured with small-scale business forms. Studies have shown that women entrepreneurs tend to have fewer competencies and thus face various challenges to develop their businesses due to the cultural, social, and economic aspects of their operations (Hendratmi et al., 2022; Kochar et al., 2022). However, even when female entrepreneurs have the required competencies, growth outcomes tend to be unpredictable (Ladge et al., 2019). Previous research has highlighted the limited understanding of the relationship between entrepreneurial competence and firm growth, especially among women entrepreneurs (Dawa et al., 2020). This research is able to complement the research conducted by Dawa et al. (2020) in explaining the role of Entrepreneurial competencies on business success, especially in SMEs. Entrepreneurial competencies are able to prepare women leaders in developing their businesses by utilizing internal strengths and absorbing external strengths that can be internalized in the business they run.

Differences in age and city are known to have no significant effect on moderating women’s leadership in successful entrepreneurship. Thus, it can be considered that it is not a key factor between age and city differences because they have the same tendency, as shown from the results above that these two moderating variables do not have a significant effect. These results also showed that economic growth in Indonesia is quite evenly distributed in every city and the age difference is not important in running a business for women, especially in the context of MSMEs.

## Conclusion

This research concludes that woman’s leadership requires absorptive capacity and entrepreneurial competencies in improving their business performance. It will have an impact on the success of MSME businesses in Indonesia. The government on a macro scale needs to pay attention to providing understanding or special training for women MSME entrepreneurs who are trying to build their business from scratch. Business challenges for women entrepreneurs are heavier than for men because there are several social, value, and cultural barriers that require special treatment and strategies in developing the potential of women entrepreneurs in Indonesia.

## Data availability statement

The raw data supporting the conclusions of this article will be made available by the authors, without undue reservation.

## Author contributions

All authors listed have made a substantial, direct, and intellectual contribution to the work, and approved it for publication.

## References

[B1] Adnani Q. E. S. Gilkison A. McAra-Couper J. (2022). A historical narrative of the development of midwifery education in Indonesia. *Women Birth.* [Epub ahead of print]. 10.1016/J.WOMBI.2022.06.007 35739017

[B2] Asandimitra N. Kautsar A. (2017). Financial self-efficacy on women entrepreneurs success. *Int. J. Acad. Res. Bus. Soc. Sci.* 7 293–300. 10.3389/fpsyg.2021.668875 34093367 PMC8170095

[B3] Ataei P. Karimi H. Ghadermarzi H. Norouzi A. (2020). A conceptual model of entrepreneurial competencies and their impacts on rural youth’s intention to launch SMEs. *J. Rural Stud.* 75 185–195. 10.1016/J.JRURSTUD.2020.01.023

[B4] Badan Pusat Statistik (2019). *Berita Resmi Statistik.* Indonesia: Badan Pusat Statistik.

[B5] Bamiatzi V. Jones S. Mitchelmore S. Nikolopoulos K. (2015). The role of competencies in shaping the leadership style of female entrepreneurs: The case of North West of England, Yorkshire, and North Wales. *J. Small Bus. Manag.* 53 627–644. 10.1111/JSBM.12173

[B6] Bibu N. A. Sala D. C. Alb M. (2016). Specific and common features in fast-growing companies from the timis̨ county (Romania). *Procedia Soc. Behav. Sci.* 221 49–56. 10.1016/J.SBSPRO.2016.05.089

[B7] Bruce R. Cavgias A. Meloni L. Remígio M. (2022). Under pressure: Women’s leadership during the COVID-19 crisis. *J. Dev. Econ.* 154:102761. 10.1016/J.JDEVECO.2021.102761 34785851 PMC8581441

[B8] Chatterjee I. Shepherd D. A. Wincent J. (2022). Women’s entrepreneurship and well-being at the base of the pyramid. *J. Bus. Ventur.* 37:106222. 10.1016/J.JBUSVENT.2022.106222

[B9] Dawa S. Namatovu R. Mulira F. Kyejjusa S. Arinaitwe M. Arinaitwe A. (2020). Entrepreneurial competences and growth of female-owned enterprises: The mediation role of absorptive capacity. *Int. J. Gend. Entrepreneurship* 13 30–49. 10.1108/IJGE-02-2020-0028/FULL/XML

[B10] Dawa S. Namatovu R. Mulira F. Kyejjusa S. Arinaitwe M. Arinaitwe A. (2021). Entrepreneurial competences and growth of female-owned enterprises: The mediation role of absorptive capacity. *Int. J. Gend. Entrep*. 13, 30–49. 10.1108/IJGE-02-2020-0028

[B11] Desky H. Mukhtasar Istan M. Ariesa Y. Dewi I. B. M. Fahlevi M. Abdi M. N. (2020). Did trilogy leadership style, organizational citizenship behaviour (OCB) and organizational commitment (OCO) influence financial performance? Evidence from pharmacy industries. *Syst. Rev. Pharm.* 11 297–305. 10.31838/srp.2020.10.50

[B12] Dheer R. J. S. Li M. Treviño L. J. (2019). An integrative approach to the gender gap in entrepreneurship across nations. *J. World Bus.* 54:101004. 10.1016/J.JWB.2019.101004

[B13] Discua Cruz A. Hamilton E. Campopiano G. Jack S. L. (2022). Women’s entrepreneurial stewardship: The contribution of women to family business continuity in rural areas of Honduras. *J. Fam. Bus. Strategy* 100505. 10.1016/J.JFBS.2022.100505

[B14] Eger C. Fetzer T. Peck J. Alodayni S. (2022). Organizational, economic or cultural? Firm-side barriers to employing women in Saudi Arabia. *World Dev.* 160:106058. 10.1016/J.WORLDDEV.2022.106058

[B15] Fahlevi M. Alharbi N. S. (2021). “The used of technology to improve health social security agency services in Indonesia,” in *3rd International Conference on Cybernetics and Intelligent Systems, ICORIS 2021*, (Indonesia: IEEE). 10.1109/ICORIS52787.2021.9649649

[B16] Fahlevi M. Aljuaid M. Saniuk S. (2022). Leadership style and hospital performance: empirical evidence from Indonesia. *Front. Psychol.* 13:911640. 10.3389/fpsyg.2022.911640 35719462 PMC9204628

[B17] Fahlevi M. Rita, Siti Rabiah A. Aristianto Pradipta I. Marta A. Dipo F. (2020). “Tourism and absorption of the labor force in Indonesia: a strategy for development,” in *5th International Conference on Energy, Environmental and Information System, ICENIS 2020*, Vol. 202 eds Warsito B. Sudarno P. T. T. (Les Ulis: EDP Sciences). 10.1051/e3sconf/202020216001

[B18] Figueroa-Domecq C. de Jong A. Williams A. M. (2020). Gender, tourism & entrepreneurship: A critical review. *Ann. Tour. Res.* 84:102980. 10.1016/J.ANNALS.2020.102980

[B19] Fine C. Sojo V. (2019). Women’s value: Beyond the business case for diversity and inclusion. *Lancet* 393 515–516. 10.1016/S0140-6736(19)30165-5 30739677

[B20] Franczak J. Margolis J. (2022). Women and great places to work: Gender diversity in leadership and how to get there. *Organ. Dyn.* 100913. 10.1016/J.ORGDYN.2022.100913

[B21] Guzman J. Kacperczyk A. (2019). Gender gap in entrepreneurship. *Res. Policy* 48 1666–1680. 10.1016/J.RESPOL.2019.03.012

[B22] Hair J. Hollingsworth C. L. Randolph A. B. Chong A. Y. L. (2017). An updated and expanded assessment of PLS-SEM in information systems research. *Ind. Manag. Data Syst.* 117 442–458. 10.1108/IMDS-04-2016-0130

[B23] Halilem N. de Silva M. Amara N. (2022). Fairly assessing unfairness: An exploration of gender disparities in informal entrepreneurship amongst academics in business schools. *Technol. Forecast. Soc. Change* 174:121295. 10.1016/J.TECHFORE.2021.121295

[B24] Handaragama S. Kusakabe K. (2021). Participation of women in business associations: A case of small-scale tourism enterprises in Sri Lanka. *Heliyon* 7:e08303. 10.1016/J.HELIYON.2021.E08303 34778588 PMC8577111

[B25] Hendratmi A. Agustina T. S. Sukmaningrum P. S. Widayanti M. A. (2022). Livelihood strategies of women entrepreneurs in Indonesia. *Heliyon* 8:e10520. 10.1016/J.HELIYON.2022.E10520 36119879 PMC9478358

[B26] IWAPI (2022). *IWAPI Ikatan Wanita Pengusaha Indonesia.* Available online at: https://iwapi.id/iwapi/index_noLogin.zul

[B27] Kamuri S. (2021). Creating as an entrepreneurial competence, innovation and performance of value-system actors in Kenya’s leather industry. *Sci. Afr.* 11:e00664. 10.1016/J.SCIAF.2020.E00664

[B28] Khalid S. Bhatti K. (2015). Entrepreneurial competence in managing partnerships and partnership knowledge exchange: Impact on performance differences in export expansion stages. *J. World Bus.* 50 598–608. 10.1016/J.JWB.2015.01.002

[B29] Kim E. Park H. (2018). Perceived gender discrimination, belief in a just world, self-esteem, and depression in Korean working women: A moderated mediation model. *Womens Stud. Int*. Forum 69, 143–150. 10.1016/j.wsif.2018.06.006

[B30] Kochar A. Nagabhushana C. Sarkar R. Shah R. Singh G. (2022). Financial access and women’s role in household decisions: Empirical evidence from India’s national rural livelihoods project. *J. Dev. Econ.* 155:102821. 10.1016/J.JDEVECO.2022.102821 35241869 PMC8856924

[B31] Ladge J. Eddleston K. A. Sugiyama K. (2019). Am I an entrepreneur? How imposter fears hinder women entrepreneurs’ business growth. *Bus. Horiz.* 62 615–624. 10.1016/J.BUSHOR.2019.05.001

[B32] Leong C. Tan F. T. C. Tan B. Faisal F. (2022). The emancipatory potential of digital entrepreneurship: A study of financial technology-driven inclusive growth. *Inf. Manag.* 59:103384. 10.1016/J.IM.2020.103384

[B33] Leung X. Y. Sun J. Asswailem A. (2022). Attractive females versus trustworthy males: Explore gender effects in social media influencer marketing in Saudi restaurants. *Int. J. Hosp. Manag.* 103:103207. 10.1016/J.IJHM.2022.103207

[B34] Lind D. A. Marchal W. G. Wathen S. A. (2018). *Statistical Techniques in Business & Economics*, 17th Edn. New York, NY: McGraw Hill Education.

[B35] Liu Y. Wei S. Xu J. (2021). COVID-19 and women-led businesses around the world. *Financ. Res. Lett.* 43:102012. 10.1016/J.FRL.2021.102012 34803532 PMC8596885

[B36] Maity S. Barlaskar U. R. (2022). Women’s political leadership and efficiency in reducing COVID-19 death rate: An application of technical inefficiency effects model across Indian states. *Socio Econ. Plan. Sci.* 82:101263. 10.1016/J.SEPS.2022.101263 35165491 PMC8828287

[B37] Mamun A. A Muhammad N. M. N. Bin Ismail M. (2017). Absorptive capacity, innovativeness and the performance of micro-enterprises in Malaysia. *Vis. J. Bus. Perspect.* 21 243–249. 10.1177/0972262917716729

[B38] Mitchelmore S. Rowley J. (2013). Entrepreneurial competencies of women entrepreneurs pursuing business growth. *J. Small Bus. Enterp. Dev.* 20 125–142. 10.1108/14626001311298448/FULL/XML

[B39] Namatovu R. Dawa S. (2017). Motivation, competences and the moderating role of business discontinuance on the entrepreneurial effect of self-employment. *Int. J. Entrepreneurship Small Bus.* 30 410–426. 10.1504/IJESB.2017.081949 35009967

[B40] Pawirosumarto S. Sarjana P. K. Gunawan R. (2017). The effect of work environment, leadership style, and organizational culture towards job satisfaction and its implication towards employee performance in Parador hotels and resorts, Indonesia. *Int. J. Law Manag.* 59 1337–1358. 10.1108/IJLMA-10-2016-0085

[B41] Prasetyo A. Putri Harwijayanti B. Ikhwan M. N. Lukluil Maknun M. Fahlevi M. (2022). Interaction of internal and external organizations in encouraging community innovation. *Front. Psychol.* 13:903650. 10.3389/fpsyg.2022.903650 35874349 PMC9301264

[B42] Pritadrajati D. S. Kusuma A. C. M. Saxena S. C. (2021). Scarred for life: Lasting consequences of unemployment and informal self-employment: An empirical evidence from Indonesia. *Econ. Anal. Policy* 70 206–219. 10.1016/J.EAP.2021.02.009

[B43] Ramadani V. Rahman M. M. Salamzadeh A. Rahaman M. S. Abazi-Alili H. (2022). Entrepreneurship education and graduates’ entrepreneurial intentions: Does gender matter? A multi-group analysis using AMOS. *Technol. Forecast. Soc. Change* 180:121693. 10.1016/J.TECHFORE.2022.121693

[B44] Saleemi S. Kofol C. (2022). Women’s participation in household decisions and gender equality in children’s education: Evidence from rural households in Pakistan. *World Dev. Perspect.* 25:100395. 10.1016/J.WDP.2022.100395

[B45] Sharif K. (2019). Transformational leadership behaviours of women in a socially dynamic environment. *Int. J. Organ. Anal.* 27 1191–1217. 10.1108/IJOA-12-2018-1611/FULL/PDF

[B46] Sheerin C. Garavan T. (2022). Female leaders as ‘Superwomen’: Post-global financial crisis media framing of women and leadership in investment banking in UK print media 2014–2016. *Crit. Perspect. Account.* 86:102307. 10.1016/J.CPA.2021.102307

[B47] Varela-Candamio L. Calvo N. Novo-Corti I. (2018). The role of public subsidies for efficiency and environmental adaptation of farming: A multi-layered business model based on functional foods and rural women. *J. Clean. Prod.* 183 555–565. 10.1016/J.JCLEPRO.2018.02.109

[B48] Vo X. V. (2016). Foreign investors and corporate risk taking behavior in an emerging market. *Financ. Res. Lett.* 18 273–277. 10.1016/j.frl.2016.04.027

[B49] Webster N. A. Haandrikman K. (2017). Thai women entrepreneurs in Sweden: Critical perspectives on migrant small businesses. *Womens Stud. Int. Forum* 60 17–27. 10.1016/J.WSIF.2016.11.001

[B50] Wilson S. Newstead T. (2022). The virtues of effective crisis leadership: What managers can learn from how women heads of state led in the first wave of COVID-19. *Organ. Dyn.* 51:100910. 10.1016/J.ORGDYN.2022.100910 35342206 PMC8940566

